# Molecular mechanisms of tumour development in glioblastoma: an emerging role for the circadian clock

**DOI:** 10.1038/s41698-024-00530-z

**Published:** 2024-02-20

**Authors:** Nina Nelson, Angela Relógio

**Affiliations:** 1 Institute for Systems Medicine and Faculty of Human Medicine, MSH Medical School Hamburg, Hamburg, 20457 Germany; 2 Institute for Theoretical Biology (ITB), Charité—Universitätsmedizin Berlin, Corporate Member of Freie Universität Berlin, Humboldt-Universität zu Berlin, and Berlin Institute of Health, Berlin, 10117 Germany; 3 Molecular Cancer Research Center (MKFZ), Medical Department of Haematology, Oncology, and Tumour Immunology, Charité—Universitätsmedizin Berlin, Corporate Member of Freie Universität Berlin, Humboldt-Universität zu Berlin, and Berlin Institute of Health, Berlin, 10117 Germany

**Keywords:** Molecular medicine, Cancer

## Abstract

Glioblastoma is one of the most lethal cancers with current therapeutic options lacking major successes. This underlines the necessity to understand glioblastoma biology on other levels and use these learnings for the development of new therapeutic concepts. Mounting evidence in the field of circadian medicine points to a tight interplay between disturbances of the circadian system and glioblastoma progression. The circadian clock, an internal biological mechanism governing numerous physiological processes across a 24-h cycle, also plays a pivotal role in regulationg key cellular functions, including DNA repair, cell cycle progression, and apoptosis. These processes are integral to tumour development and response to therapy. Disruptions in circadian rhythms can influence tumour growth, invasion, and response to treatment in glioblastoma patients. In this review, we explore the robust association between the circadian clock, and cancer hallmarks within the context of glioblastoma. We further discuss the impact of the circadian clock on eight cancer hallmarks shown previously to link the molecular clock to different cancers, and summarize the putative role of clock proteins in circadian rhythm disturbances and chronotherapy in glioblastoma. By unravelling the molecular mechanisms behind the intricate connections between the circadian clock and glioblastoma progression, researchers can pave the way for the identification of potential therapeutic targets, the development of innovative treatment strategies and personalized medicine approaches. In conclusion, this review underscores the significant influence of the circadian clock on the advancement and understanding of future therapies in glioblastoma, ultimately leading to enhanced outcomes for glioblastoma patients.

## Introduction

Glioblastoma (GBM) is one of the most mortal cancers with a 12-15 months median survival and 90% recurrence after therapy^1^. Current standard and experimental therapies have failed so far to significantly improve GBM outcome mainly due to its heterogeneity and plasticity^2^. GBM is characterized by a high level of molecular diversity^3^. It is subdivided into three major molecular subtypes: mesenchymal-like, classical-like, and proneural-like that rather represent plastic states on a continuum, influenced by the tumour microenvironment^3^.

Due to unsatisfactory success rates of current therapeutic regimes in GBM treatment, new approaches are urgently needed. Notably, disruption of the molecular clock is associated with cancer^4^. For instance, meta-analyses showed a positive correlation between breast cancer risk and shift work^5^, as well as between prostate and breast cancer risk and late eating behaviour^6^. Therapeutic approaches targeting the molecular clock (chronotherapy) delivered promising results in cell and mouse models, as well as in clinical studies in different cancer entities^7^. In GBM, the limited number of clinical studies which analysed the optimal treatment timepoint for TMZ (Temozolomide) reported conflicting results in terms of patient survival and adverse effects^1^. However, pre-clinical models were hopeful and may result in a more comprehensive landscape of understanding clinical results in the future^1^. It is thus important to attain a deep insight into the molecular mechanisms regarding the GBM-clock interplay in order to optimize future study designs.

**Fig. 1:**
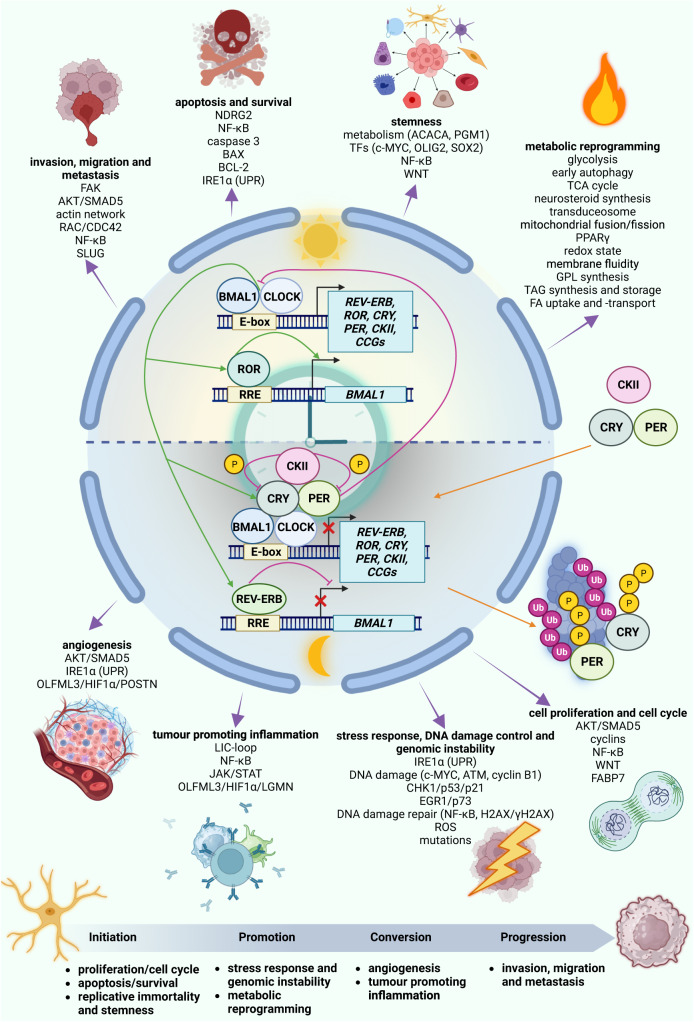
Overview of the core-clock network and structure of the review. The figure shows an overview of the core clock network. The components of this network are interconnected by transcriptional and translational feedback loops. Briefly, CLOCK (Circadian locomoter output cycles protein kaput)/BMAL1 (Basic helix-loop-helix ARNT-like protein 1) heterodimers activate the transcription of target genes like *PER (PERIOD)* (*PER1,2,3)*, *CRY (CRYPTOCHROME)* (*CRY1,2)*, *REV-ERB (Nuclear Receptor Subfamily 1 group D)* (*REV-ERBα,β*), and *ROR (RAR (Retinoic Acid Receptor) related Orphan Receptor)* (*RORα,β,γ*) via binding to E-boxes on their promotor regions. CRY and PER proteins inhibit CLOCK/BMAL1. ROR and REV-ERB proteins compete for the binding to RORE elements on the promotor of *BMAL1* to activate (ROR) or inhibit (REV-ERB) the transcription of *BMAL1*^9^. The clock regulates hallmarks of cancer in GBM, in particular cell proliferation and cell cycle, apoptosis and survival, stemness, stress response, DNA-damage control and genomic instability, metabolic reprogramming, angiogenesis, tumour promoting inflammation and migration, invasion and metastasis, which are crucial for cancer progression. The pathways impacted by the clock are listed beneath each hallmark and will be discussed in further detail throughout this review. The figure was created with BioRender.com.

The circadian clock drives the oscillating expression of genes and other cellular processes in a 24-h rhythm, enabling an optimal adjustment of the organism to the natural dark:light cycle. It comprises a central pacemaker in the suprachiasmatic nucleus (SCN) and peripheral clocks in the individual cells and is crucial for maintaining body homoeostasis. The circadian clock controls almost all physiological processes and its disruption is linked to diseases such as cancer and metabolic disorders^8^. The clock is driven by a core set of elements, the core-clock, interconnected in translational and transcriptional feedback/feed-forward loops^9^ (Fig. 1).

This review explores the molecular mechanisms of the interplay between the circadian clock and GBM. We will concentrate on eight important cancer hallmarks known to be regulated by the circadian clock (Fig. 1)^10^. Cancer hallmarks encompass a set of common principles that are crucial for the multistep-development from a healthy cell to a malignant cancer cell. They were recently reviewed and updated by Douglas Hanahan and the reader is referred to this review for a more detailed overview^11^. We will also cover the expression and clinical relevance of circadian clock proteins and the level of disturbance of the circadian rhythm in GBM, and discuss current chronotherapeutic treatment approaches, which may provide interesting therapy options in the future.

## Impact of the circadian clock on GBM cell proliferation and cell cycle

The proliferation of GBM cells was shown to be associated with GBM subtype and -subtumour localization. GBM cells of the proneural-like and classical-like subtype are characterized by ectopic activation of PDGFRA (Platelet-Derived Growth Factor Receptor Alpha) respectively EGFR (Epithelial Growth Factor Receptor). They show a higher abundance of proliferating cells compared to GBM cells of the mesenchymal-like subtype^12,13^. Another important hallmark of glioblastoma to promote proliferation was shown to be the hyperactivation of the PI3K (Phosphatidylinositol 3-kinase)/AKT (Ak-strain Thyoma)/mTOR (mammalian Target Of Rapamycin) pathway^14^.

**Fig. 2:**
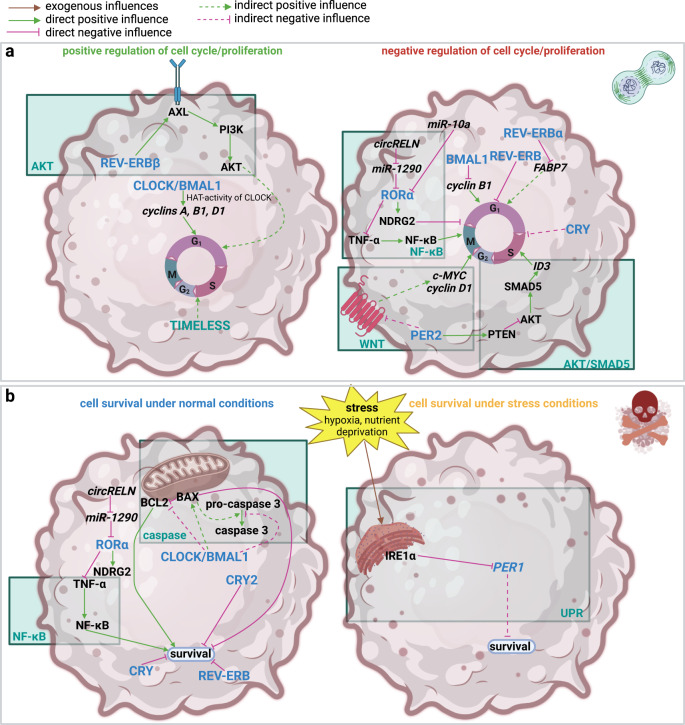
Impact of the circadian clock on proliferation and cell cycle, apoptosis and survival in GBM. **a** Impact of the circadian clock on proliferation and cell cycle in GBM. Cell proliferation in GBM is regulated by different pathways, which are under clock control, namely the PI3K/AKT, the NF-κB and the WNT pathway. In particular, the PI3K/AKT pathway is frequently altered in GBM. The cell cycle can be subdivided into four phases: G_1_, S, G_2_ and M. Cell cycle progression, especially the passing of checkpoints to initiate the cell cycle and to progress from one phase to the next is tightly controlled in healthy cells. However, it is deregulated in tumours due to tumour-specific alterations, which allows for unrestricted hyperproliferation. REV-ERBβ activates the PI3K/AKT pathway as it increases the transcription and membrane localization of AXL. AKT is a known promotor of cell cycle progression. The HAT (Histone-Acetyl Transferase) activity of CLOCK is implicated in the transcription of cyclins A, B1 and D1. The HAT activity of CLOCK allows it to regulate gene expression at the level of chromatin remodelling. It is essential for upholding a functional circadian rhythm and enhanced by dimerization of CLOCK with BMAL1. TIMELESS was also shown to have a positive effect on cell cycle progression. PER2 activates PTEN and therefore inhibits the AKT/SMAD5/ID3-dependent cell cycle progression. It also inhibits the WNT pathway. WNT activates c-MYC and cyclin D1 to promote cell cycle progression. RORα is a negative regulator of cell cycle progression. It activates NDRG2 and it inactivates the TNFα/NF-κB pathway. RORα is often repressed in GBM by means of *miR-10a* and the *circRELN/miR-1290* axis. REV-ERB inhibits cell cycle progression, in the case of REV-ERBα by transcriptional repression of *FABP7*. BMAL1 represses the transcription of *cyclin* B1. **b** Impact of the circadian clock on apoptosis and survival in GBM. Resistance to cell death is another important cancer hallmark. Apoptosis can be induced either by external factors or by the inner stress status of the cell such as DNA damage. These stressors lead to the cleavage of inactive pro-caspases to active caspases and downstream apoptosis-promoting pathways. Tumour cells typically achieve the capability to survive even under stress conditions. The circadian clock influences GBM cell survival both under stressed and normal conditions. Under normal conditions RORα is a negative regulator of survival. It activates NDRG2 and it inactivates the TNFα/NF-κB pathway. It is repressed by *circRELN/miR-*1290. CRY and REV-ERB also negatively regulate cell survival. CLOCK/BMAL1 block the activation of pro-caspase 3. They inactivate the apoptosis-promoting protein BAX (BCL-2 (B-Cell Lymphoma 2)-associated X protein) and activate the apoptosis-inhibiting protein BCL-2 (B-cell Lymphoma 2). BAX can be activated either by the intrinsic or the extrinsic apoptosis pathway. It is inactivated by BCL-2. BAX leads to the release of cytochrome C from the mitochondria, which in turn leads to the cleavage of pro-caspase 3. Under stress the UPR gets activated which induces the UPR protein IRE1α and consequently reduces the expression of *PER1* to promote survival. The UPR is activated by high amounts of unfolded proteins in the rER (rough endoplasmic reticulum) and leads to the activation of the intrinsic apoptosis pathway. The figure was created with BioRender.com.

In GBM, core-clock proteins influence cell cycle progression and proliferation in a differential manner (Fig. 2a).

Ectopic expression of CLOCK/BMAL1, TIMELESS, and REV-ERBβ were shown to have a positive influence on proliferation^15–23^. In more detail, CLOCK increased the expression of *cyclins A, B1*, and *D1* due to its histone transferase activity and activated the NF-κB (Nuclear Factor kappa B) pathway. Its expression was de-repressed in GBM by downregulation of *miR-124 (microRNA-124)*^17,19^. Notably, a study performed by Dong et al. found that downregulation of *CLOCK/BMAL1* negatively affected proliferation and cell cycle progression in Glioblastoma Stem Cells (GSCs) but not in Neural Stem Cells (NSCs)^16^. REV-ERBβ was shown to positively regulate the transcription and plasma membrane localization of the receptor tyrosine kinase AXL (AXL receptor tyrosine kinase) and its downstream targets PI3K and AKT to promote cell cycle transition. Of note, KD (knock-down) of *REV-ERBβ* only led to cell cycle arrest and inhibition of proliferation in GBM cells but not in human astrocyte (HA) control cells^23^. Interestingly, REV-ERBα was found to negatively influence cell proliferation in U251 GBM cells by repression of *FABP7 (Fatty Acid Binding Protein 7)*, which is a known crucial regulator of adult neurogenesis^24^. In addition, treatment of GBM cells with the REV-ERB agonists SR9009 or SR9011 reduced proliferation^16,21^. Also, RORα, PER2, and CRY inhibited proliferation by different mechanisms such as downregulation of the NF-κB, WNT (Wingless/Integrated) and PI3K/AKT pathways (see Fig. 2a)^16,25–30^. Intriguingly, BMAL1 was also shown to negatively regulate cell cycle progression and proliferation in different GBM cell lines, as well as in an orthotopic mouse A530 model^31–33^.

Therefore, the effect of the clock on GBM cell proliferation appears to be complex and dependent on clock protein isoforms, as well as on the cell lines and experimental conditions utilized. Seemingly contradictory findings were reported for BMAL1, which was shown to act both as an oncogene, as well as a tumour suppressor. This dual function of BMAL1 on carcinogenesis was also observed for other cancer entities such as colorectal cancer^34^.

The fact that cancer cells seem to be more sensitive to a disruption of clock genes than healthy cells with regard to proliferation^16,23^ may make them a very promising target for cancer therapy. In general, CRY and REV-ERB agonists were unanimously successful in inhibiting proliferation in primary GSCs, a good model system for patient heterogeneity. It would therefore be interesting to explore those agonists in a larger cohort of patient-derived systems in the future, together with a deeper analysis of their subtype specificity, and the possible influence of intra-tumour heterogeneity and genomic alterations.

## Impact of the clock on GBM apoptosis and survival

GBM is marked by deregulation of the p53 (protein of 53 kDa) pathway in 84% of GBM patients, which allows GBM cells to effectively escape apoptosis. Deregulation of this pathway occurs in connection to p53, MDM2 (Mouse Double Minute 2 homolog), and ARF (ADP Ribosylation Factor). It is one of the core pathways affected by GBM^2^.

Disruption of the molecular clock was shown to promote tumour cell survival by different mechanisms (Fig. 2b). CLOCK/BMAL1 heterodimers generally increased the survival of GBM cells and GSCs in vitro and in vivo^15,16,20,35^. In patient-derived GSCs KD or KO (knock-out) of *CLOCK/BMAL1* or treatment of the cells with CRY or REV-ERB agonists known to negatively affect the CLOCK/BMAL1 heterodimer (SR9009, SR9011, KL001) induced apoptosis only in GSCs, but not in NSC control cells^16^. In contrast, Gwon et al. showed that overexpression of *BMAL1* in U87-MG cells induced apoptosis^31^. On the other hand, RORα increased apoptosis by inactivation of the TNFα (Tumour Necrosis Factor alpha)/NF-κB axis, and activation of NDRG2 (N-Myc Downstream-Regulated Gene 2)^25,27^ It was reduced by the *circRELN (circular RNA Reelin) /miR-1290 (microRNA-1290)* axis in GBM^26^. SHP656 was shown to selectively activate CRY2 and treatment with SHP656 had a negative effect on cell viability. Notably, this effect was stronger in GSCs compared to differentiated GBM cells. This may suggest that clock control of apoptosis might depend on the differentiation status of the GBM cell^36^. Interestingly, stress-induced activation of the UPR (Unfolded Protein Response) protein IRE1α (Inositol-Requiring Enzyme 1 alpha) led to the downregulation of *PER1* in U87-MG cells and to the maintenance of GBM cell survival under stress^37^.

In summary, CLOCK/BMAL1 had a positive effect on GBM cell survival in most studies whereas CRY2, PER1, and RORα negatively affected survival. Interestingly, *PER1* downregulation by the UPR was implicated in survival under stress conditions, which may point to a selective survival advantage of GBM cells under stress.

Although the number of studies on the glioblastoma-clock interplay with regard to apoptosis are limited there are some interesting studies with therapeutical relevance performed in patient-derived GSCs. Especially the study by Dong et al. showed the selective effect of CRY/REV-ERB agonists on GSCs, but not NSCs and also showed tumour-selective effects of those treatments on other cancer hallmarks^16^. Also, the study performed by Miller et al. may have important consequences for targeted therapy, as KL001 would be expected to have stronger effects in patients with high CRY2/CRY1 ratios^36^.

## Impact of the clock on GBM replicative immortality and stemness

Replicative immortality is an important feature of cancers and is achieved through overactivation or ectopic expression of TERT (Telomerase)^38^. *TERT* promotor mutation status is an important biomarker for the classification of GBM, and *TERT* is upregulated in 80% of GBM cases^2,39^. Interestingly, GBM-characteristic mutations of *TERT* were found to be associated with a more aggressive phenotype and a poorer prognosis^2^. In Mouse Embryonic Fibroblasts (MEFs) *TERT* showed rhythmic expression patterns and was transcriptionally regulated by CLOCK/BMAL1, and reduction of CLOCK was associated with shorter telomere length^40^. This opens the possibility that the deregulation of CLOCK/BMAL1 may also be responsible for conferring replicative immortality in GBM.

GSCs constitute an important component of the GBM cell population and are associated with treatment resistance and GBM recurrence. It is a topic of debate if GSCs could be defined as a distinct cell population or rather as a state on a continuum spanning from more differentiated to more stem-cell resembling states. This was described as the concept of the plasticity of GBM cells^3^. CLOCK/BMAL1 were found to act as positive regulators of stemness by means of activation of the NF-κB pathway, and by the direct transcriptional regulation of the stemness-promoting factors *c-MYC (cellular proto-oncogene MYC), OLIG2 (Oligodendrocyte Transcription Factor 2)*, and *SOX2 (SRY-Box Transcription Factor 2)*^16,19^. Interestingly, a study conducted by Chen et al., which utilized KDs of *CLOCK* and *BMAL1* or treatment with SR9009/SR9011 found that CLOCK/BMAL1 promoted stemness in GSCs via the induction of *ACACA (Acetyl-CoA Carboxylase Alpha)* and *PGM1 (Phosphoglucomutase 1)*. This finding linked stemness to glucose metabolism and fatty acid synthesis^15^. On the other hand, PER2 and RORα were found to reduce stemness^25,27,29^.

Hence, the possibility of conferring GBM replicative immortality by a deregulated clock may be interesting to pursue in future studies. Given the positive influence of CLOCK/BMAL1 on stemness and the clinical implications associated with GSCs, which are dormancy and therapeutic resistance, therapies should focus on reducing the levels and/or activity of the CLOCK/BMAL1 complex.

**Fig. 3:**
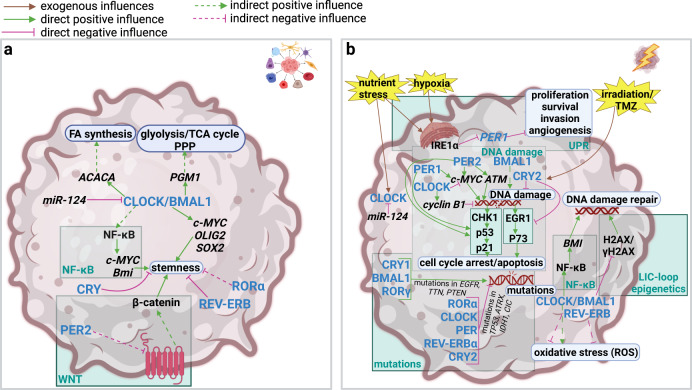
Impact of the circadian clock on stress response, DNA damage control and genomic instability and stemness in GBM. **a** Impact of the circadian clock on stemness in GBM. Stemness is regulated by transcriptional programs. Cancer stem cells represent a major problem in therapy because they are often resistant to treatment and are responsible for residual disease and cancer recurrence. CLOCK/BMAL1 activate the transcription of *ACACA* and *PGM1 s*pecifically in GBM cells, which links stemness to fatty acid synthesis, glycolysis, the TCA cycle and the pentose phosphate pathway (PPP). They are also implicated in the transcription of, or positively influence, several stemness-promoting transcription factors, specifically *c-MYC*, NF-κB, *SOX2* and *OLIG2*. CRY, REV-ERBβ and RORα inhibit stemness. PER2 reduces stemness by inhibition of the WNT pathway. WNT activates the transcription factor β-catenin to promote stemness. **b** Impact of the circadian clock on stress response, DNA damage control and genomic instability in GBM. The circadian clock is implicated in responses to DNA damage inducers (radiotherapy, TMZ), as well as responses to oxidative and nutrient stress and hypoxia. DNA damage is induced by TMZ or irradiation. Healthy cells typically respond to DNA damage by cell cycle arrest or apoptosis. These responses are regulated by a set of proteins, most notably p53. p53 is abolished or inactivated in 84% of GBM patients, which allows them to avoid cell cycle arrest and apoptosis following DNA damage. DNA damage is promoted by PER1, PER2 and BMAL1 whereas CLOCK and CRY2 inhibit it. In more detail, PER1 and PER2 induce *c-MYC*, which hyperactivation leads to DNA damage, whereas CLOCK reduces *c-MYC*. PER2 also positively influences *ATM* (*Ataxia Telangiectasia Mutated)*. Following DNA damage cell cycle arrest and apoptosis is achieved by two different pathways: the CHK1 (Checkpoint Kinase 1)/p53/p21 (Protein of 21 kDa) pathway is inhibited by CLOCK and activated by PER1 and PER2; the EGR (Early Growth Response 1)/p73 (Protein of 73 kDa) pathway is inhibited by CRY2. CLOCK/BMAL1 promotes DNA damage repair by the NF-κB/*Bmi (BMI1 Polycomb Ring Finger Proto-Oncogene)* and the H2AX (Histone 2 AX)/γH2AX (gamma Histone 2 AX) pathways. CLOCK is implicated in the response to nutrient stress and is repressed by *miR-124* and *PER1* is reduced by the UPR-protein IRE1α in response to hypoxia and nutrient stress. Clock proteins are also implicated in DNA mutations, and regulate both their abundance and specificity. *CRY1*, *BMAL1*, and *RORγ* are associated with a higher mutation rate and mutation hotspots in *EGFR*, *TTN*, and *PTEN* genes. *RORα*, *PER1*, *PER2*, *PER3*, *CLOCK*, *REV-ERBα*, and *CRY2* are associated with a lower mutation rate and mutations skewed towards *TP53*, *ATRX*, *IDH1*, and *CIC*. In particular, a high mutation rate respectively high genomic instability is associated with cancer. Mutations in *EGFR, PTEN, TP53* and *IDH1* are highly common in GBM. The figure was created with BioRender.com.

Also, the interplay between stemness and metabolism may link certain alterations of the clock to niches requiring adaptions in energy metabolism and stem-cell-like phenotypes alike. It may be possible that those three alterations mutually reinforce each other (Fig. 3a).

We would expect from these results that a combined approach of targeting clock components and energy metabolism may have additive effects in patient subgroups with strong alterations in both pathways. GSCs differ in their metabolic phenotype from differentiated GBM cells i.e., they depend more strongly on oxidative phosphorylation^41^, which could make the mitochondria a possibly more interesting target than glucose transporters or glycolysis enzymes. Drugs targeting glucose i.e., HK2 (Hexokinase 2) or amino acid i.e., GLS-1 (Glutaminase 1) or arginine auxotrophy metabolism or the Krebs cycle i.e., IDH-1 (Isocitrate Dehydrogenase) are currently in early clinical trials^2^.

## Impact of the clock on stress response, DNA damage control and genomic instability

GBM tumours display a typical spatial organization consisting of a hypoxic core and a less dense tumour periphery. The tumour core is marked by a high amount of stress from hypoxia and nutrient deficiency to which GBM cells have to adapt. The hypoxia-reactive phenotype residing in the tumour core was classified as predominantly mesenchymal-like and represented several adaptations such as glycolysis, quiescence, genomic instability, and upregulation of programs associated with migration and invasion^13^.

The clock machinery was shown to modulate the response to stressors leading to DNA damage. Those stressors included irradiation, TMZ and oxidative stress. It was also shown to be implicated in the adaption to nutrient and hypoxic stress (Fig. 3b). The function of CLOCK/BMAL1 in oxidative stress regulation and DNA damage repair under normal conditions i.e., without external stress induction is controversial in experimental studies^17,19–21^. In a computational study using TCGA (The Cancer Genome Atlas) glioma data, *CRY1*, *BMAL1*, and *RORγ* were associated with a higher mutation rate and mutation hotspots in *EGFR*, *TTN (Titin)*, and *PTEN (Phosphatase and Tensin Homologue)* genes. *RORα*, *PER1*, *PER2*, *PER3*, *CLOCK*, *REV-ERBα*, and *CRY2* were associated with a lower mutation rate and mutations were skewed towards *TP53*, *ATRX (Alpha Thalassemia/Mental Retardation Syndrome X-Linked)*, *IDH1*, and *CIC (Capicua Transcriptional Repressor)*^42^.

Under stress inducing DNA damage (TMZ treatment, radiation) clock genes are highly implicated in the modulation of stress resistance. Irradiation was shown to induce different clock proteins: PER2 was induced by irradiation to promote DNA damage and apoptosis, and *PER2* KD reduced the radiosensitivity of tumours in vivo using an U343 mouse model^43^. Interestingly, apoptosis induction after irradiation was highest at the peak of PER2 expression in rat glioma, but not in healthy brain tissue^44^. Of note, also PER1 was upregulated by irradiation and led to increased radiosensitivity in U343 cells^45^. However, in a rat glioma model there was no significant difference in apoptosis induction after irradiation at the peak of PER1 expression compared to the trough of PER1 both in glioma and normal brain tissue^44^. A study conducted by Fan et al. using a rat glioma model showed upregulation of CRY2 and loss of rhythmicity after irradiation only in glioma but not in normal brain tissue. This indicated as for PER2^44^ that glioma and normal brain cells seemed to adjust their clock differently in response to irradiation^46^. At the timepoint of maximal CRY2 expression proliferation was highest and apoptosis was lowest. The opposite was found when CRY2 levels were lowest. Interestingly, such a correlation between radiosensitivity and CRY2 levels was only seen in glioma, but not healthy rat brain tissue^46^. Similar to CRY2, CLOCK also prevented DNA damage-induced apoptosis and -cell cycle arrest following irradiation in U87-MG cells^47^. On the other hand, DNA damage and subsequent apoptosis were highest at the peak of *BMAL1* expression in mouse MES-GBM cells (astrocytes with mutations in *NF1 (Neurofibromin 1)* and *TP53* serving as a model for the mesenchymal-like GBM subtype) when treated with TMZ^48^.

Nutrient stress and hypoxia are known to induce response pathways such as the UPR and cancer cells hijack the UPR to adapt to external stress and to promote survival^49^. PER1 was found to be implicated in UPR and CLOCK in the response to serum starvation^19,37^.

Based on the above-described reports, clock genes were shown to play a major role in regulating DNA damage response and apoptosis after treatment with DNA-damaging agents (irradiation, TMZ). This provides an interesting opportunity to use pharmacological manipulation of clock genes or treatment time to optimize treatment sensitivity. Such an approach would be especially useful as it was shown for the two clock proteins CRY2 and PER2 that the effects of their expression on radiosensitivity were exclusively observed in glioma, but not in healthy brain tissues. Regarding the role of CLOCK/BMAL1 in DNA damage repair, oxidative stress and mutation frequency/genomic instability, the conflicting results published so far may be the results of different experimental systems, or data used. Also, the studies presenting data on the function of CLOCK/BMAL1 on ROS (Radical Oxygen Species) induction did not use direct genetic manipulation of *CLOCK/BMAL1*, but indirect manipulation by pharmacological treatments, which may have increased off-target effects.

A very interesting finding is the deregulation of the circadian clock by irradiation and subsequent loss of rhythmicity^43–46^. This could implicate that GBMs with a rather healthy circadian rhythm may show stronger sensitivity to radiotherapy than GBMs with an already disrupted circadian rhythm. Also, the studies presented on TMZ and irradiation showed an effect of treatment time in the models used^44,46,48^ These are promising findings for chronotherapy studies in humans, given that both treatment forms are already established in the current standard-of-care protocol.

## Impact of the clock on GBM metabolic reprogramming

GBM cells show characteristic metabolic alterations associated with other cancer hallmarks such as invasion and tumour-promoting inflammation. Those comprise (1) the establishment of a Warburg-phenotype population with high uptake of glucose, however, some GBM cells especially GSCs also use oxidative phosphorylation for energy production, (2) high uptake and metabolism of glutamine for nucleotide and amino acid synthesis, and (3) high uptake of cholesterol, elevated sterol synthesis and increased fatty acid and triglyceride metabolism and -synthesis. Alterations of energy metabolism are driven by the TME (Tumour Microenvironment). In this context, astrocytes produce cholesterol and glutamine, which are taken up by the tumour cells. Further, the brain-specific environment with elevated levels of glucose compared to other organs and tumour-cell intrinsic changes promote this metabolic phenotype^41^.

**Fig. 4:**
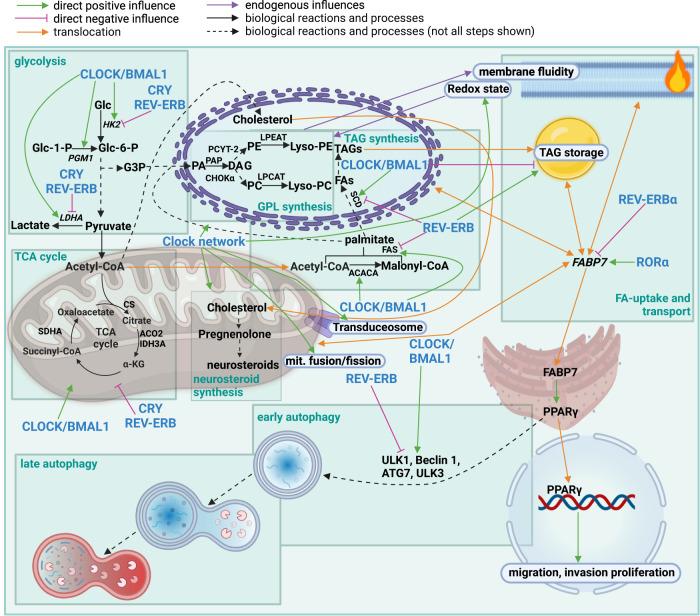
Impact of the circadian clock on metabolic reprogramming in GBM. The circadian clock impacts major metabolic pathways, which are important for GBM progression such as glycolysis and lipid metabolism, as well as early autophagy. Glycolysis is a pathway which converts glucose to pyruvate. It produces ATP and NADH for energy and biomolecule production. HK2 is a key enzyme of glycolysis. It traps glucose in the cell in the form of glucose-6-phosphate (Glc-6-P). Pyruvate can be converted to Acetyl-CoA (Acetyl-Co-enzyme A) and utilized in the TCA-cycle or converted to lactate. The conversion to lactate is achieved by LDHA. LDHA is one of the key enzymes of aerobic glycolysis (Warburg phenotype). This pathway is often preferred by cancer cells over oxidative phosphorylation even in the presence of oxygen to provide intermediates for amino acid and nucleotide production. The TCA-cycle consists of several oxidative decarboxylation steps to produce redox equivalents and ATP for later oxidative phosphorylation. It also serves as a hub for the production of various biomolecules. CLOCK/BMAL1 positively influence several glycolysis enzymes (*PGM1, HK2*) and the TCA cycle. They also act as positive transcriptional regulators of *LDHA* to promote aerobic glycolysis. CRY and REV-ERB show a negative influence on both glycolysis (*HK2*), *LDHA* and the TCA cycle. REV-ERBα inhibits the transcription of *FABP7* and RORα activates it. FABP7 is implicated in FA (Fatty Acid) uptake and -transport and FA-loaded FABP7 activates the transcription of *PPARγ (Peroxisome Proliferator Activated Receptor Gamma)* to promote migration, invasion and proliferation. CLOCK/BMAL1 are important positive regulators of pathways, which are crucial for tumour cells, but less for healthy cells (autophagy and de-novo FA synthesis). Specifically, CLOCK/BMAL1 positively influence FAS (Fatty Acid Synthetase), SCD (Stearoyl-CoA Desaturase) and ACACA, whereas REV-ERB negatively influences SCD and FAS. FA synthesis originates from Acetyl-CoA, which is converted to Malonyl-CoA by ACACA. FAS then merges Acetyl-CoA with Malonyl-CoA to form palmitate, which is transported into the smooth endoplasmic reticulum (sER). Palmitate is processed to FAs in a multistep process involving SCD. They can be further converted to TAGs (Triacylglycerol) by addition of glycerol. Autophagy is a process in which the cell removes dysfunctional or no-longer necessary components. It consists of early autophagy in which the autophagosome is formed from the rER and late autophagy in which the autophagosome is fused with the lysosome to form the autolysosome. CLOCK/BMAL1 positively regulates the early autophagy proteins ULK1 (Unc-51 (Uncoordinated-51) Like Autophagy Activating Kinase 1), Beclin1, ATG7 (Autophagy Related 7) and ULK3 (Unc-51 Like Autophagy Activating Kinase 3), whereas REV-ERB negatively influences them. The clock network as a whole regulates several metabolic pathways i.e., neurosteroid synthesis from cholesterol, mitochondrial fusion and fission, mitochondrial transport (transduceosome), GPL synthesis and the redox state of the cell. Neurosteroids are formed from cholesterol. Cholesterol is produced from AcetylCoA in the sER. It is converted to pregnenolone and further to neurosteroids in the mitochondria. GPLs are produced from the glycolysis intermediate glyceraldehyde-3-phosphate (G3P), which is converted into phosphatidic acid (PA) in the sER. PA is converted to diacyl-glycerol (DAG) by PAP (Prostatic Acid Phosphatase). DAG serves as an intermediate to form either Lyso-PE (Phosphatidylethanolamine) or Lyso-PC (Phosphatidylcholine). PCYT-2 (Phosphate Cytidylyltransferase 2) converts DAG to PE. PE is then converted to Lyso-PE by LPEAT (Lyso-Phosphatidylethanolamine Acyltransferase). On the other hand, DAG can be converted to PC by CHOKα (Choline Kinase Alpha) and then to Lyso-PC by LPCAT (Lyso-Phosphatidylcholine Acyltransferase). GPLs regulate the fluidity of the plasma membrane and their synthesis is regulated by the redox state of the cell. The figure was created with BioRender.com.

The clock network was shown to be implicated in the regulation of glycolysis, TCA-cycle (Tricarboxylic Acid cycle), lipid metabolism, neurosteroid synthesis, mitochondrial fusion/fission, and autophagy (Fig. 4).

**Fig. 5:**
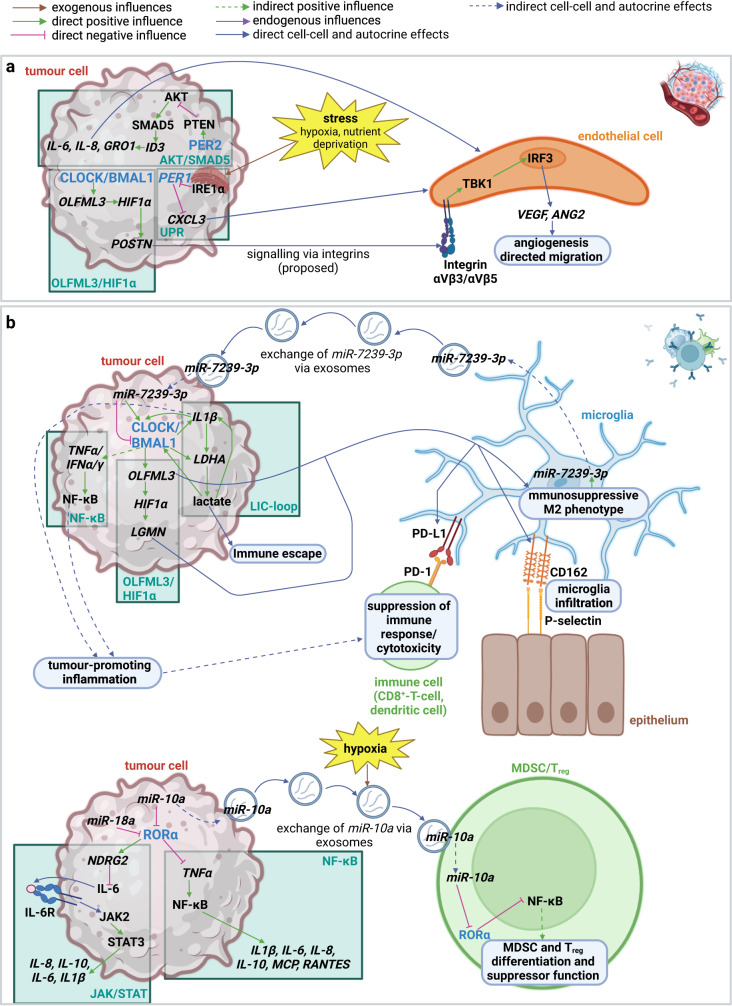
Impact of the clock on angiogenesis and tumour promoting inflammation in GBM. **a** Impact of the clock on GBM angiogenesis. CLOCK/BMAL1 activate the transcription of POSTN via the CLOCK/BMAL1-OLFML3-HIF1α axis. POSTN promotes angiogenesis induction in endothelial cells via activation of TBK1 possibly by integrins, which is proposed to activate the transcription factor IRF3 (Interferon Regulatory Factor 3) leading to the expression of pro-angiogenic factors and directed migration of endothelial cells. Stress of hypoxia or nutrient deficiency activates the UPR-protein IRE1α, which in turn reduces the expression of *PER1*. PER1 acts as a transcriptional repressor of the pro-angiogenic factor *CXCL3*. PER2 activates PTEN and therefore inhibits the AKT/SMAD5/ID3-dependent transcription of the angiogenesis-promoting cytokines *IL-6, IL-8* and *GRO-1*. Angiogenesis-promoting factors affect endothelial cells to form new blood vessels. This process is especially important for tumour cells after reaching a certain size to ensure sufficient supply of oxygen and nutrients. **b** Impact of the clock on GBM tumour promoting inflammation. Tumour promoting inflammation refers to the reprogramming of immune cells to immunosuppressive and tumour-promoting phenotypes. In the context of GBM especially microglia, MDSCs and regulatory T-suppressor cells (T_regs_) are regulated by the circadian clock. Microglia are brain-resident macrophages. Tumour promoting inflammation in the context of microglia is promoted by CLOCK/BMAL1, which induces the migration and immunosuppressive phenotype of microglia, as well as secretion of tumour-promoting mediators into the microenvironment. In more detail, CLOCK/BMAL1 activate the TNFα/NF-κB pathway and the LIC-loop to promote tumour promoting inflammation and immune escape. The LIC-loop is a feedforward loop in which CLOCK/BMAL activates the transcription of *IL1β* and *LDHA* leading to the production of lactate. IL1β positively influences *CLOCK/BMAL1* and *LDHA* and lactate positively influences *CLOCK/BMAL1* and *IL1β*. CLOCK/BMAL1 induces the reprogramming of microglia via the HIF1α/OLFML3/LGMN axis. Reprogrammed microglia suppress immune cells, promote tumorigenesis and acquire an infiltrative phenotype. Reprogrammed microglia also signal back to the tumour cell by exosomes containing *miR-7239-3p* to increase *CLOCK* and reduce *BMAL1*. RORα negatively affects tumour promoting inflammation in MDSCs and T_regs_ via inhibiting the secretion of tumour promoting cytokines into the TME, as well as inhibiting their differentiation and function as suppressor cells. It acts by activating NDRG2, which in turn reduces the JAK2/STAT3-dependent expression of *IL-6*. It also negatively regulates the TNFα/NF-κB-dependent expression of various cytokines associated with immune responses. RORα is negatively regulated by *miR-10a* and *miR-18a*. The figure was created with BioRender.com.

Using patient-derived GSCs and either KDs of *BMAL1* or *CLOCK* or treatment with SR9009, SR9011 or KL001, it was found that CLOCK/BMAL1 showed differential occupation of E-box sites within the genome between NSCs and GSCs. Those sites, unique for GSCs, were associated with glycolysis, TCA cycle, oxidative phosphorylation and lipid biosynthesis^16^. Further, CLOCK/BMAL1 controlled lactate synthesis via the Lactate-IL1β-CLOCK loop (LIC-loop). The LIC-loop is a feed-forward loop, which links energy metabolism to the clock and the immune system and is represented in detail in Fig. 5b. The feed-forward nature of this loop underscores the importance of CLOCK/BMAL1 in promoting a Warburg-phenotype^17^. In addition, in GSC272 cells CLOCK was found to positively regulate *PGM1*, which converts Glc-1-P to Glc-6-P thereby increasing the glycolytic flux^15^. The function of the clock on lipid synthesis and metabolism is complex. Of note, a functional clock was needed for GPL (glycerophospholipid) synthesis, regulation of membrane fluidity, and neurosteroid synthesis^50,51^. Clock proteins further controlled de-novo fatty acid synthesis, fatty acid transport, and lipid storage as shown in Fig. 4^15,21,24,52^.

A study by Sulli et al. performed in A172 GBM cells found that SR9009/SR9011 specifically targeted pathways, which were crucial for cancer cells, but less so for healthy cells, in particular de-novo fatty acid synthesis and early autophagy^52^.

Thus, the clock network was shown to be implicated in several metabolic processes that are particularly critical for tumour cells. These comprised aerobic glycolysis, autophagy, and de-novo fatty acid synthesis. Those pathways may therefore render feasible targets for therapeutic approaches such as treatment with SR9009 or SR9011^16,52^.

Two key studies underline the strong potential of the energy metabolism-clock interplay for glioblastoma targeting. The study by Dong et al. used patient-derived GSCs as a model system and showed the differential occupation of metabolic gene promotors by CLOCK/BMAL1 in GSCs and NSCs, some in key genes of the Warburg phenotype, in particular *HK2* and *LDHA (Lactate Dehydrogenase A)*^16^. The study by Sulli et al. showed the specific targeting of crucial cancer pathways especially autophagy and de-novo fatty acid synthesis by REV-ERB and CRY agonists, but it remains to be seen if similar effects will be observed in patient-derived systems^52^. This means that cancer populations relying heavily on one or more of those sensitive metabolic pathways, e.g., highly proliferating Warburg-type tumour cells could profit heavily from therapies involving clock manipulation.

## Impact of the clock on GBM angiogenesis

Angiogenesis is known as an important hallmark of GBM: GBM is a highly vascularized tumour due to its high proliferation rate and subsequent need for oxygen and nutrients. The tumour microvasculature is a critical supporting pillar for tumour cell infiltration into surrounding brain areas, one of the main problems in GBM therapy^53^. The expression of *BMAL1* was shown to be positively correlated with the pro-angiogenic factors *HIF1α (Hypoxia Induced Factor 1 alpha)*, *VEGF (Vascular Endothelial Growth Factor)*, and *ANG2 (Angiopoietin 2)* in a cohort of 79 patients of high- and low-grade glioma (HGG and LGG). HGG tumours showed significantly increased levels of BMAL1, HIF1α, VEGF, and ANG2 compared to LGG tumours or healthy tissue^54^. Pang et al. found that downregulation of *BMAL1* or *CLOCK* in GSC cell lines significantly reduced angiogenesis in vitro and in orthotopic C57BL/6 mouse models^55^. Mechanistically, the CLOCK/BMAL1-OLFML3 (Olfactomedin Like 3)-HIF1α axis was found to be responsible for the upregulation of angiogenesis. POSTN (Periostin) promoted angiogenesis by inducing the transcription and activation of TBK1 (TANK Binding Kinase 1)^55^. In contrast, PER2 was shown to be a negative regulator of angiogenesis due to its negative impact on the AKT dependent expression of angiogenesis promoting cytokines^30^. Stress such as hypoxia and nutrient deprivation was also shown to contribute to angiogenesis. In U87-MG cells, stress induced the activation of the UPR protein IRE1α, which in turn reduced *PER1*. PER1 was shown to act as a transcriptional repressor for the angiogenesis-promoting chemokine *CXCL3 (C-X-C Motif Chemokine Ligand 3)*. Therefore, this signalling pathway led to the adaption of GBM cells to hypoxia/nutrient deprivation-induced stress via the upregulation of tumour angiogenesis^37^.

In summary, deregulation of the clock promoted angiogenesis. Whereas CLOCK/BMAL1 had a positive influence on angiogenesis PER proteins acted as negative regulators of angiogenesis (Fig. 5a).

The key study by Pang et al. could show a transcriptional effect of CLOCK/BMAL1 on the cytokine axis to promote angiogenesis also in an in vivo context and a negative influence of SR9009 on angiogenesis^55^. Given that angiogenesis poses such an important hallmark of GBM this is a very promising finding. Due to the positive significant correlation between an altered clock i.e., increased levels of BMAL1 in GBM, and VEGF levels^54^ future studies may combine a VEGF inhibitor such as bevacizumab with a clock manipulator such as a CRY or REV-ERB agonist. Bevacizumab is approved for recurrent GBM, however only showed a positive effect on PFS, but not on OS^56^. The response to bevacizumab may be enhanced by this combination strategy.

Also, as described in the following chapter the same cytokine axis involved in angiogenesis also plays a role in tumour-promoting inflammation, another crucial GBM hallmark. Targeting this axis would therefore potentially benefit tumours with different microenvironments i.e., highly vascularized and highly immune cell infiltrated areas alike.

## Impact of the clock on GBM tumour promoting inflammation

Tumour-promoting inflammation, meaning the reprogramming of immune cells within the tumour microenvironment to an immunosuppressive tumour-promoting phenotype, is a crucial component of tumorigenesis. Tumour cells recruit immune cells, and the crosstalk between reprogrammed immune cells and tumour cells promotes important tumour traits such as survival and EMT (Epithelial to Mesenchymal Transition)^57^. GBM is known for its highly immunosuppressive nature. TAMs (Tumour Associated Macrophages) i.e., microglia cells and bone-marrow-derived macrophages form the major part of the GBM microenvironment^58^. They constitute up to 50% of the whole tumour mass^58^. MDSCs (Myeloid-Derived Suppressor Cells) comprise another major pillar of immunosuppression in GBM^59^. The immune-cell infiltrated phenotype of GBM is particularly associated with the mesenchymal-like subtype and is driven by loss of *NF1*^60^.

Clock proteins mainly control the immune cell microenvironment via the activity of CLOCK/BMAL1 and RORα (Fig. 5b). CLOCK/BMAL1 induces tumour promoting inflammation whereas RORα abrogates it. High levels of *BMAL1* and low levels of *RORα* are associated with the recruitment of suppressor cells and immune cell infiltration^42^. CLOCK/BMAL1 was shown to be involved in microglia reprogramming to a tumour-promoting and immunosuppressive phenotype, and the secretion of tumour-promoting cytokines and chemokines^15,17,35^. RORα was associated with the inhibition of MDSC differentiation and function, and the suppression of tumour-promoting cytokine secretion^25,27,28^.

Mechanistically, CLOCK/BMAL1 were found to directly activate the transcription of the chemokine *OLFML3* and indirectly activate the transcription of the chemokine *LGMN (Legumain)* via the OLFML3/HIF1α axis in GBM cells. Both chemokines promoted the infiltration of microglia into the TME and induced a shift in microglial phenotype towards an immunosuppressive/tumour-promoting phenotype^35^. Additional molecular mechanisms contributing to the promotion of an immunosuppressive TME by CLOCK/BMAL1 were described as well (see Fig. 5b)^15,17^.

Of note, tumour-promoting microglia were also found to influence the expression of clock proteins in GBM cells, mainly the downregulation of *BMAL1* by tumour-promoting microglia exosomes carrying *miR-7239-3p (microRNA-7239-3p)* and concurrent upregulation of *CLOCK*^32^. This impressively demonstrated the interdependence of tumour and immune cells.

On the other hand, *RORα* was found to be downregulated in GBM and silenced by *miR-10a (microRNA-10a)*^28^ and *miR-18a (microRNA-18a)*^27^. In GBM, RORα reduced tumour-promoting inflammation by downregulation of the JAK2 (Janus Kinase 2)/STAT3 (Signal Transducer And Activator Of Transcription 3) /IL-6 (Interleukin 6) and the TNF-α/NF-κB pathway^25,27^. Interestingly, *miR-10a* was also shown to be exchanged with MDSCs via exosomes and to repress *RORα* in these cells. This led to the promotion of the differentiation of myeloid cells to MDSCs, as well as to the development of their suppressor function^28^. This was more pronounced under hypoxia and may explain the high level of tumour-promoting inflammation in hypoxic areas of the tumour enriched in tumour cells of the mesenchymal-like subtype^3,61^.

The studies conducted so far revealed the importance of clock genes in the regulation of the immune tumour microenvironment. As a large amount of research work has already been performed in the field of tumour immunotherapy in GBM without reaching major break-throughs^62^, it may be interesting to combine therapeutic strategies targeting the clock with immune therapies with the aim to improve the efficiency of stand-alone tumour immunotherapy.

The studies by Xuan et al.^35^ and Li et al.^32^ notably underlined the complex interplay of the immune-tumour cell crosstalk and the clock, and it would be very interesting to further explore this research area and to continue to investigate both the effects of immune system on tumour cells and vice versa, as well as their link to the clock. Generally, the most aggressive mesenchymal-like phenotype is linked to the highest level of immune infiltration, as well as to tumour recurrence and this suggests reducing tumour-promoting inflammation may be one of the crucial fields to tackle. Xuan et al. found a significant effect of SR9009 in reducing tumour promoting inflammation and enhancing anti-tumour immunity in GSC cell lines^35^, but this should be further investigated in other model systems. Also, two studies found evidence for exosomes in mediating the interplay of the clock and tumour promoting inflammation, which makes them another interesting target^28,32^. As reviewed by Pinheiro et al.^63^ and Dain et al.^64^ there are different possibilities to reprogram immune cells such as cytokine therapy and immune checkpoint inhibitors. Approaches targeting IFNγ (Interferon gamma), TNFα, or the PD-1 (Programmed Cell Death 1)/PDL-1 (Programmed Cell Death Ligand 1) axis may provide excellent opportunities for combination with clock-based strategies, as those targets are controlled by the clock^63^. In addition, strategies targeting tumour antigens, namely vaccines, oncolytic viruses, and CAR-T-cells (Chimeric Antigen Receptor T-cells) may exploit differences in the clock and clock-controlled genes between GBM and healthy cells^63,64^.

Exosomes are currently being explored as diagnostic tools. In the context of the clock, they may be used to diagnose the two relevant mi-RNAs *miR-10a* and *miR-7239-3p* that are exchanged via exosomes. Furthermore, GBM-derived exosomes are used as a vehicle to selectively target drugs to the exosome homing-site^65^. Together with optimized drug timing this strategy may help to further reduce toxicity to healthy cells and enhance anti-tumour effects of the drug.

## Impact of the clock on GBM invasion, migration and metastasis

In GBM, cell motility is especially important for the infiltration of adjacent brain areas whereas distant metastases are rare^66^. Infiltration poses a serious problem for therapy as infiltrated areas cannot be completely removed by surgery without destroying critical brain structures^67^.

**Fig. 6:**
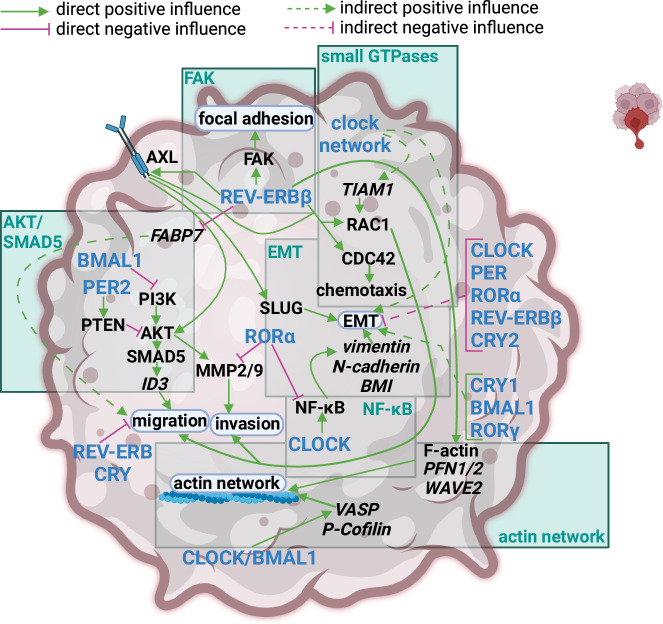
Impact of the circadian clock on cell motility in GBM. Migration, invasion, chemotaxis, and EMT are affected by the molecular clock. Migration and invasion enable cancer cells to form local or distant metastasis. Especially invasion allows them to cross through the extracellular matrix, which is a crucial step for metastasis. Chemotaxis is referred to as the sensing of certain signals, so-called chemo-attractants, which enable directed migration and invasion. EMT is a transcriptional program regulated by a set of master transcription factors. It enables cancer cells to acquire a mesenchymal and an infiltrative phenotype. REV-ERBβ positively affects cell motility by increasing the expression and membrane localization of the receptor tyrosine kinase AXL. AXL induces the EMT master transcription factor SLUG (Neural crest transcription factor Slug Protein snail (*D. melanogaster* Snail zinc-finger transcriptional repressor) homolog 2), as well as AKT, CDC42 (Cell Division Cycle 42) and RAC to promote migration, invasion and chemotaxis. REV-ERBβ can also induce CDC42 and RAC independent on AXL. Further, it has a positive impact on FAK (Focal Adhesion Kinase) and positively influences the actin network, but it has a negative impact on *FABP7*-dependent migration. Focal adhesion as induced by FAK enables cells to tightly anchor themselves to the extracellular matrix, which induces several downstream signalling pathways linked to tumorigenesis. VASP is implicated in the elongation of actin filaments and P-Cofilin in the severing of F-actin filaments. Hence, both proteins are important regulators of the actin network dynamic. This dynamic is important for cell motility. PER2 inhibits AKT-dependent migration and invasion via activation of PTEN. CLOCK/BMAL1 are positive regulators of the actin network. They induce the expression of *VASP (Vasodilator-stimulated phosphoprotein)* and *P-Cofilin*. BMAL1 is a negative regulator of AKT-dependent migration and invasion. CLOCK positively influences NF-κB-dependent EMT. Other clock proteins are also implicated in the regulation of EMT, but the mechanism is unclear. The clock network as a whole has a positive influence on EMT. The clock network also controls the rhythmicity of the RAC-GEF TIAM1 and therefore of the migration capacity of the tumour cell. GEFs (Guanine Exchange Factors) are activators of small GTPases such as RAC. The figure was created with BioRender.com.

In GBM, both EMT^68^ and the levels of the RAC-GEF (Ras-Related C3 Botulinum Toxin Substrate 1 Guanine Exchange Factor) TIAM1 (T Cell Lymphoma Invasion And Metastasis 1), but not the levels of RAC1, were found to be rhythmic^69^ indicating an interplay between circadian rhythm and GBM motility. Clock proteins were found to regulate GBM migration, invasion, chemotaxis, the actin cytoskeleton network, focal adhesion, and EMT (Fig. 6). CLOCK/BMAL1 was reported to regulate the actin cytoskeleton components VASP (Vasodilator Stimulated Phosphoprotein), and P-Cofilin via the LIC loop^17^, and to exhibit a positive effect on cell motility^16,20^. Interestingly, studies focusing solely on BMAL1 reported a negative effect on cell migration and invasion^31,32,70^. On the other hand, CLOCK positively influenced invasion and EMT^18,19^. In contrast, RORα and PER2 were observed to inhibit migration and invasion^26,29,30^. Notably, REV-ERBα and REV-ERBβ showed opposite effects on GBM cell migration, invasion, and chemotaxis as outlined in Fig. 6^23,24^.

A computational study found a positive influence of *CRY1*, *BMAL1*, and *RORγ* on EMT whereas *CLOCK*, *PER1/2/3*, *RORα*, *REV-ERBβ*, and *CRY2* negatively influenced EMT. This study contradicted many of the experimental results; however, it should be considered that the authors used TCGA data for HGG and LGG^42^.

Based on the results described above, a connection of GBM cell motility and circadian rhythm is evident. Given that GBM infiltration is the main cause for recurrence and mortality this topic extremely relevant for future studies^67^. For instance, REV-ERBβ may be a very interesting target as its KD strongly affected the motility of GBM cells, but not human astrocytes^23^. In future studies, this could be analyzed in patient-derived cells. Therapeutically, Dong et al. showed that CRY and REV-ERB agonists reduced the migration of patient-derived GSCs again underlying the huge potential for those drugs in targeting GBM hallmarks in a patient-derived model^16^.

## Clinical applications of the clock in glioma prognosis and therapy

### Expression and rhythm of clock proteins in glioma and their implication on disease progression

The regulation of the expression of clock proteins and their impact on clinical outcome is complex and probably context-dependent. The studies published so far showed both oncogenic and tumour suppressor functions of BMAL1, CLOCK, PER1, PER2, PER3, CRY2, and REV-ERBβ. However, higher evidence for an oncogenic function was found for BMAL1 and CLOCK and higher evidence for a tumour suppressor function was found for PER2 and PER3, consistent with their role in the positive respectively negative arm of the clock network. Surprisingly, different isoforms of the same gene seemed to function differently in glioma progression (Table 1).

**Table 1:** Overview of the expression of clock protein and their correlation to clinical parameters

	Oncogene	Tumour suppressor
BMAL1	79 glioma patients/controls: significantly higher expression of BMAL1 in glioma^54^ TCGA-LGG/HGG: high expression of *BMAL1* was associated with higher grade and shorter OS^42^ TCGA-LGG/HGG: *BMAL1* expression significantly higher in HGG compared to LGG^22^ IC injection of patient-derived GSCs in NSG mice: KD of *BMAL1* reduced tumour growth and increased the survival of the mice^16^ TCGA-LGG/GBM: higher expression of *BMAL1* in GBM compared to LGG, high expression of *BMAL1* was associated with a poorer prognosis^16^ U87-MG cells: Overexpression of *IDHmut* (R132H) decreased the expression of BMAL1 compared to overexpression of *IDH WT*^97^ TCGA glioma: high expression of *BMAL1* was associated with a shorter OS^71^ 622 glioma samples and 628 healthy controls: higher expression of *BMAL1* in glioma^98^	IC injection of A530 cells in C57BL/6 mice: KO of *BMAL1* increased tumour growth^33^ TCGA-LGG/GBM: expression of *BMAL1* lower in LGG compared to healthy controls but no significant differences between GBM and healthy controls^97^
CLOCK	TCGA-LGG/HGG: 2.5% of patients showed copy number gains of *CLOCK* gene^42^ TCGA-LGG/GBM: 5% of patients showed copy number gains of *CLOCK* gene in GBM and 2.8% in LGG^15^ IC injection of GSC272 cells in SCID mice: KD of *CLOCK* increased survival^15^ IC injection of patient-derived GSCs in NSG mice: KD of *CLOCK* reduced tumour growth and increased the survival of the mice^16^ TCGA-LGG/GBM: higher expression of *CLOCK* in GBM compared to LGG, high expression of *CLOCK* was associated with a poorer prognosis^16^. U87-MG cells: Overexpression of *IDHmut* (R132H) decreased the expression of CLOCK compared to overexpression of *IDH WT*^97^. 16 glioma tissue samples and two healthy brain control tissues, GBM cell lines U87-MG, T98G, A172, U251 and glia cell lines HASP and HEB: higher expression of CLOCK in glioma tissue and GBM cell lines^19^. 60 glioma patients/controls: high expression of CLOCK correlated with shorter OS^18^. 67 patients, comparison of tumour and normal brain tissue from the same patient: CLOCK was upregulated in HGG compared to LGG samples and in tumour tissue compared to healthy brain tissue^99^. 622 glioma samples and 628 healthy controls: *CLOCK* SNP rs7698022 correlated with a poor prognosis in HGG^98^ 622 glioma samples and 628 healthy controls: higher expression of *CLOCK* in glioma^98^	TCGA-LGG/HGG: high expression of *CLOCK* was associated with lower grade and longer OS^42^. TCGA-LGG/HGG: *CLOCK* expression significantly lower in HGG compared to LGG^22^ TCGA glioma: high expression of *CLOCK* was associated with a longer OS^71^.
PER1	U87-MG cells: Overexpression of *IDHmut* (R132H) decreased the expression of PER1 compared to overexpression of *IDH WT*^97^. 622 glioma samples and 628 healthy controls: *PER1* SNP rs2289591 correlated with a poor prognosis in HGG^98^ 622 glioma samples and 628 healthy controls: higher expression of *PER1* in glioma^98^	TCGA-LGG/HGG: high expression of *PER1* was associated with lower grade and longer OS^42^. TCGA-LGG/HGG: *PER1* expression significantly lower in HGG compared to LGG^22^ IC injection of A530 cells in C57BL/6 mice: PER1 levels were lower in the tumour than in non-tumour brain tissue^33^. 33 glioma tissue samples/healthy controls: PER1 levels lower in glioma tissue compared to healthy tissue and in HGG compared to LGG^100^ TCGA glioma: high expression of *PER1* was associated with a longer OS^71^ TCGA glioma: high expression of *PER* correlated with longer OS in astrocytic gliomas but not in oligodendrogliomas independent of IDH mutational status and was an independent prognostic factor^101^.
PER2	U87-MG cells: Overexpression of *IDHmut* (R132H) decreased the expression of PER2 compared to overexpression of *IDH WT*^97^.	TCGA-LGG/HGG: high expression of *PER2* was associated with lower grade and longer OS^42^. TCGA-LGG/HGG: *PER2* expression significantly lower in HGG compared to LGG^22^ TCGA-LGG/GBM: high expression of *PER2* was associated with a better prognosis^16^. TCGA/CCGA glioma and 59 glioma patient samples/controls: High expression of PER2 was correlated with lower grading and a better prognosis^30^. IC injection of U87-MG cells into nude mice: overexpression of *PER1* reduced tumour growth and increased OS^30^. 33 glioma tissue samples/healthy controls: PER2 levels lower in glioma tissue compared to healthy tissue, no difference between HGG and LGG^100^ TCGA glioma: high expression of *PER2* was associated with a longer OS^71^. 92 glioma tissues vs. healthy controls: downregulation of *PER2* in glioma via promotor methylation^102^. TCGA glioma: high expression of *PER* correlated with longer OS in astrocytic gliomas but not in oligodendrogliomas independent of IDH mutational status and was an independent prognostic factor^101^.
PER3	U87-MG cells: Overexpression of *IDHmut* (R132H) decreased the expression of PER3 compared to overexpression of *IDH WT*^97^.	TCGA-LGG/HGG: 1% of patients showed deletions of *PER3* gene^42^. TCGA-LGG/HGG: high expression of *PER3* was associated with lower grade and longer OS^42^. TCGA-LGG/HGG: *PER3* expression significantly lower in HGG compared to LGG^22^ TCGA-LGG/GBM: high expression of *PER3* was associated with a better prognosis^16^. TCGA glioma: high expression of *PER3* was associated with a longer OS^71^ 622 glioma samples and 628 healthy controls: lower expression of *PER3* in glioma^98^. TCGA glioma: high expression of *PER* correlated with longer OS in astrocytic gliomas but not in oligodendrogliomas independent of IDH mutational status and was an independent prognostic factor^101^.
CRY1	TCGA-LGG/HGG: high expression of *CRY1* was associated with higher grade and shorter OS^42^. TCGA-LGG/HGG: *CRY1* expression significantly higher in HGG compared to LGG^22^. U87-MG cells: Overexpression of *IDHmut* (R132H) decreased the expression of CRY1 compared to overexpression of *IDH WT*^97^. TCGA glioma: high expression of *CRY1* was associated with a shorter OS^71^. 622 glioma samples and 628 healthy controls: higher expression of *CRY1* in glioma^98^	
CRY2	U87-MG cells: Overexpression of *IDHmut* (R132H) decreased the expression of CRY2 compared to overexpression of *IDH WT*^97^. 69 glioma tissue/control samples: higher levels of CRY2 in HGG compared to LGG and in glioma compared to healthy tissue^103^.	TCGA-LGG/HGG: high expression of *CRY2* was associated with lower grade and longer OS^42^. TCGA-LGG/HGG: *CRY2* expression significantly lower in HGG compared to LGG^22^ TCGA-LGG/GBM: high expression of *CRY2* was associated with a better prognosis^16^. TCGA glioma: high expression of *CRY2* was associated with a longer OS^71^.
REV-ERBα		TCGA-LGG/HGG: high expression of *REV-ERBα* was associated with lower grade and longer OS^42^. TCGA-LGG/HGG: *REV-ERBα* expression significantly lower in HGG compared to LGG^22^ TCGA-LGG/GBM: high expression of *REV-ERBα* was associated with a better prognosis^16^ TCGA glioma: high expression of *REV-ERBα* was associated with a longer OS^71^.
REV-ERBβ	Glioma samples and glioblastoma cell lines U87-MG, LN-18, T98G, U118-MG, U373-MG: REV-ERBβ levels were higher in GBM samples compared to LGG samples and higher in GBM cell lines compared to human astrocytes^23^	TCGA glioma: high expression of *REV-ERBβ* was associated with a longer OS^71^.
RORα		TCGA-LGG/HGG: high expression of *RORα* was associated with lower grade and longer OS^42^. TCGA and CCGA glioma: *RORα* expression was lower in GBM compared to LGG and *RORα* is enriched in IDHmut gliomas and served as an independent prognostic factor for patient survival^27^. 70 glioma patients and 10 healthy controls: RORα levels higher in HGG than in LGG and in glioma than in healthy controls^27^. IC injection of patient-derived GSC2C and GSC4D cells in nude mice: high expression of RORα was associated with increased survival^27^.
RORγ	TCGA-LGG/HGG: high expression of *RORγ* was associated with higher grade and shorter OS^42^.	

Of note, there is evidence for a role of clock proteins in therapy resistance. Preclinical models provided evidence for a relationship between the circadian clock and radiotherapy resistance in glioblastoma. In a rat glioma model, radioresistance was highest at the timepoint of the CRY2 peak^46^ and *CLOCK* KD reduced radioresistance in U87-MG cells^47^ pointing to a promotion of radioresistance for these two clock proteins. Contrarily, PER1 and PER2 decreased resistance to radiotherapy. In more detail, radioresistance was increased when *PER2* was ablated either in a loss-of-function mouse model or an orthotopic mouse model using U343 *PER2* KD cells, and radioresistance was highest at the timepoint of the PER2 trough in a rat glioma model^43,44^. *PER1* KD in U343 cells led to increased radioresistance in vitro^45^. A computational study using data obtained from the Genomics of Drug Sensitivity in Cancer (GDSC) database reported that the expression levels of the four clock-associated genes *BMAL1, NPAS2 (Neuronal PAS Domain Protein 2)*, *DBP (D-Box Binding PAR BZIP Transcription Factor)* and *CRY2* correlated with the sensitivity of GBM to various drugs^71,72^. In particular, *BMAL1* expression positively correlated with TMZ sensitivity in vitro^71^. These results were supported by experimental findings in mouse MES-astrocytes^48^. Further, POSTN was shown to increase resistance to anti-angiogenic treatment in GBM^73^. As mentioned, *POSTN* was shown to be positively regulated by the CLOCK/BMAL1 complex^55^. Therefore, one explanation for the rather unsatisfactory response to the angiogenesis inhibitor bevacizumab in GBM^56^ could be an upregulation of *CLOCK/BMAL1*, as observed in the majority of human studies (see Table 1).

Altering the circadian rhythm was suggested to increase glioma risk in a jetlag mouse model^74^. Interestingly, the central circadian clock may be more important for GBM genesis than the internal clock of the tumour cells. A study by Wagner et al. showed that tumour growth was increased in mice when GBM A530 cells were injected at night compared to injection in the morning, but the internal clock status of the A530 cells (synchronization at different time points, but injection at the same time point) did not impact tumour outcome^33^. Another interesting study showed that in a *D. melanogaster* GBM model the circadian rhythm of the flies was lost with GBM progression and the period lengthened from 24 to 28 h. This was correlated with reduced insulin in the brain and reduced activity of the central pacemaker, which could be partially rescued by the application of external insulin. Of note, changing the dark:light cycle from 12:12 h (24 h period) to 14:14 h (28 h period) to adjust for the period lengthening observed in the GBM flies, significantly reduced tumour development^75^.

Surprisingly, whereas some studies reported that GBM cell lines/tissues exhibited disrupted circadian rhythms^46,50^ the majority of studies found normal circadian rhythms in GBM models^16,48,51,69,76^. A study by Zhang et al. using TCGA and GTEx datasets showed that the circadian rhythm, i.e., the expression of clock proteins was more severely altered in LGG than in GBM and only in LGG was the alteration of clock gene expression a prognostic factor^77^.

Hence overall, the circadian rhythm may be less severely altered in GBM than in other tumour entities. However, as shown throughout this review, altering clock proteins exhibited strong effects on GBM tumorigenesis in a variety of model systems, and the circadian rhythm of the model system (mouse, *D. melanogaster*) strongly influenced GBM progression and -risk^33,74,75^. In the following subsection, we will discuss how this knowledge may be applied in the context of GBM therapy.

### Current evidence on chronotherapy in GBM

Due to the apparent lack of current therapeutic options in GBM treatment with breakthrough success, this section will explore the potential of GBM chronotherapy as an exciting and innovative approach in this field. As briefly referred to in the introduction, chronotherapy uses the clock as a therapeutic tool. Specifically, it aims to (1) optimize the timepoint of drug administration and (2) manipulate the circadian clock by different means such as light or drugs^7^.

Temporal drug effects were found in pre-clinical models for a variety of drugs as listed in Table 2. TMZ, a currently used therapy in GBM is the most realistic candidate to study GBM chronotherapy in the near future. TMZ is rapidly absorbed and easily crosses the blood-brain-barrier. It also has a short half-life time of 1.8 h making it very suitable for chronotherapy^78^. Although pre-clinical results suggested a significant correlation between the clock and drug response^48^, there is currently no definite evidence for a benefit of timed treatment in humans^78^. Specifically, a phase II clinical trial of morning vs. afternoon administration of TMZ (*n* = 39) did not find significant differences in overall survival (OS) or adverse effects^79^, whereas a retrospective study including 166 participants found a significant benefit of morning TMZ administration on OS^80^. Similarly, a retrospective study on morning vs. evening radiotherapy (*n* = 109) failed to find significant differences in adverse effects and OS^81^, despite pre-clinical evidence of the involvement of clock proteins in radioresistance^43–47^. This may have different reasons: 1) the studies to date were limited to only 2 timepoints (morning vs. evening), and 2) published studies did not stratify for specific properties such as sex and age known to influence the time of optimal drug administration^82–85^. It is also important to keep in mind that the temporal drug response is dependent on the individual circadian profile of a patient, and thus taking a drug at a time, which is misaligned with the individual clock could potentially lead to increased toxicity and reduced response rates^86^.

**Table 2:** Overview of chronotherapy in glioblastoma

SR9009	Decreased the population of M2 microglia and microglia infiltration and immunosuppressive function in a CT2A mouse model and decreased the transwell migration of microglia treated with conditioned medium of GSC272 cells^35^. Increased the survival of C57BL/6 mice intracranially injected with CT2A cells and increased apoptosis and decreased proliferation and stemness of CT2A cells in vivo^35^. Reduced the proliferation of U251 and T98G cells^22^. Increased the survival of mice intracranially injected with GSC272 cells^15^. Reduced the stemness of patient-derived GSCs^15^. Reduced stemness and proliferation and increased apoptosis more strongly in GSCs than in NSCs and decreased TCA cycle, glycolysis, and de-novo lipid synthesis in patient-derived GSCs^16^. Decreased tube formation of mouse primary endothelial cells and iHUVECs treated with conditioned medium from mouse QPP7 GSCs and reduced angiogenesis in a GSC272 and CT2A mouse model^55^. Cytotoxic for glioblastoma cells but not healthy cells at comparable concentrations and comparable effectiveness to TMZ in a mouse glioblastoma model with minor side effects, targeted pathways of relevance for glioma but not healthy cells (autophagy, de novo lipid synthesis)^52^. Reduced cell viability and led to cell cycle arrest in T98G cells, reduced oxidative stress and increased lipid storage^21^. Highest effect at 18 h ZT and lowest effect at 6 h ZT in T98G cells^21^.
SR9011	Reduced the proliferation of U251 and T98G cells^22^. Increased the survival of mice intracranially injected with GSC272 cells^15^. Reduced the stemness of patient-derived GSCs^15^. Reduced stemness and proliferation and increased apoptosis more strongly in GSCs than in NSCs and decreased TCA cycle, glycolysis, and de-novo lipid synthesis in patient-derived GSCs, additive effects when combined with KL001^16^. Cytotoxic for glioblastoma cells but not healthy cells at comparable concentrations, targeted pathways of particular relevance for glioma but not healthy cells (autophagy, de novo lipid synthesis)^52^.
Dp44mT	Reduced the levels of RORα in a mouse glioma model and increased the survival of the mice, decreased tumour size, tumour cell proliferation, and tumour-promoting inflammation and increased apoptosis in vivo^25^.
Bortezomib (BOR, proteasome inhibitor)	In a mouse model of IC injection of A530 cells into C57BL/6 mice BOR administration at night was more effective than in the morning^33^. In T98G cells BOR response rates were dependent on treatment time with a period of 30 h which was shifted 6 h upon KD of *BMAL1*^50^. Highest effect at 24 h ZT and lowest effect at 30 h ZT in T98G cells, additive effects with SR9009^21^.
SHP656	In a mouse model of IC injection of patient-derived GSCs in NSG mice treatment with SHP656 reduced tumour growth and increased the survival of the mice^16^. Specifically targeted CRY2 and inhibited the viability of patient-derived GSCs stronger than that of differentiated GBM cells^36^.
KL001	Reduced stemness and proliferation and increased apoptosis more strongly in GSCs than in NSCs and decreased TCA cycle, glycolysis, and de-novo lipid synthesis in patient-derived GSCs, additive effects when combined with SR9011^16^.
TMZ	Treatment with TMZ showed highest response rates at the peak of *BMAL1* expression in mouse MES-astrocytes. The temporal treatment response variation was lost upon KO of *BMAL1*^48^. *BMAL1* expression levels correlated with sensitivity to TMZ treatment in TCGA data^71^.
1A-116 (RAC inhibitor)	In LN229 cells treatment with 1A-116 was more effective at the peak of *PER1*/*TIAM1* expression but this effect was lost upon KO of *BMAL1*^69^.
VX-745 (p38 MAPK inhibitor)	In IM3 cells VX-745 showed the highest effect when p38 MAPK activity was lowest in human astrocytes^76^.
curcumin	At low doses of curcumin (5 µM) C6 cells retained a functional circadian rhythm as measured by PER2-Luc. Circadian rhythms of mitosis were measured for controls and 10 µM curcumin but at 5 µM curcumin C6 cells showed an ultradian rhythm of mitosis. Circadian rhythms of apoptosis were found for controls and 5 µM curcumin and lost for 10 µM curcumin. Highest effects of curcumin were measured 6–11 h prior to the peak of PER2^104^.
Doxorubicin	Doxorubicin uptake in C6 nuclei showed a circadian rhythm and peaked 6 h before PER2 and 12 h after BMAL1. The nuclear transport protein CRM1 was also rhythmic and peaked 4 h after BMAL1^105^.
Sevoflurane	Increased levels of RORα, reduced proliferation, migration, and invasion of T98G and N18 cells and increased their apoptosis^26^.
Lectin from *Abelmoschus esculentus*	Downregulated BMAL1 and CLOCK in U87-MG cells and led to decreased proliferation and migration, and increased oxidative stress and apoptosis^20^.

Furthermore, directly targeting the circadian clock by pharmacological means was shown to be highly effective (Table 2). Notably, the small molecule clock-protein agonists SR9009, SR9011, and KL001 were shown to selectively target tumour cells. Healthy cells on the other hand were not or only minorly affected^16,52^. Exemplarily, SR9009 was shown to be as effective as TMZ in a mouse GBM model, but exhibited strongly reduced side effects^52^.

Therefore, treatments directly targeting core clock components may be very interesting to pursue in future clinical studies due to their selectivity for GBM over healthy cells^16,52^.

## Discussion

### Perspectives on the clock-GBM interplay

Throughout this review we described several mechanisms regarding the impact of the circadian clock on GBM development. Yet, also GBM may influence the circadian clock functioning, and it is thus relevant to consider this aspect for a better understanding of the clock-GBM interplay, as described below. It is known from previous studies that the clock and the hallmarks of cancer act in tight interdependence, which is why we assume such an interdependence in GBM as well^10,11^.

As alluded to in the previous sections, different *miRNAs* were shown to influence the clock and to be altered in GBM, notably *miR-10a, miR-124, miR-1290* and *miR-18a*^19,26–28^. Importantly, also *mi-RNAs* produced in the TME (i.e., *miR7239-3p* produced in immunosuppressive microglia reprogrammed by the tumour) were shown to influence the clock, which underlines the importance of the TME in this context^32^.

Circadian clock genes are regulated also at the post-translational level, by several modifications including phosphorylation, SUMOylation and O-GlcNAcylation, which impact their stability and intracellular localization^87^. Published data on these modifications in the context of GBM are still lacking. However, ERK1/2 (Extracellular Signal-Regulated Kinase 1/2) and AKT1, both highly relevant for GBM progression, were shown to negatively regulate CLOCK/BMAL1 by phosphorylation in insulin-sensitive tissues for AKT and the SCN for ERK1/2, which led to their retention in the cytosol^88,89^. However, in another study *AKT*^*-/-*^ mice showed decreased amplitudes of BMAL1 and CLOCK and increased amplitudes of REV-ERBα and PER2 in lung endothelial cells^90^. Therefore, one can assume a differential regulation of the clock by AKT depending on the tissue and possibly AKT protein level vs. levels of activated AKT. Moreover, in the SCN ERK1/2 were shown to be activated in a circadian manner by light, and to subsequently activate the expression of *PER1*^89^. Therefore, it will be interesting to explore in future work if, and to which degree, AKT1 and ERK1/2 are implicated in the regulation of the clock in GBM as well.

Interestingly, a computational study using TCGA data reported differential regulation of circadian genes in glioma by hypoxia, namely HIF1α^91^. As GBM consists of a hypoxic core and a less hypoxic peripheral area it will be very interesting to investigate spatial regulation of clock genes in this context.

It is well known that the circadian clock is a molecular network of transcriptional and translational feedback loops (see also introduction) i.e., CCGs and clock genes mutually affect each other^9^. As shown in the previous sections AKT, as well as its downstream effector HIF1α were found to be CCGs in GBM^30,55^. On the other hand, as indicated above AKT and HIF1α are known regulators of clock genes^88,91^. In GBM, which is marked by hyperactivation of the PI3K/AKT/mTOR pathway and increased HIF1α induced by oncogenes and hypoxia^2^, there would be a possibility that the assumed equilibrium of the clock genes and the GBM genes would shift towards the GBM genes, leading to a potentially deregulated clock network and promotion of GBM development. These findings may point to a mutual regulatory loop between the clock and GBM drivers, and suggest that in GBM a shift of the balance between the components of this assumed loop may happen, thus enabling an acceleration of GBM development. Certainly, an interesting hypothesis, which is worth exploring in future studies.

### Perspectives on the clock in the context of GBM therapy

Applications of circadian medicine will necessarily involve a detailed characterization of the profiles of core clock genes to allow for both stratification of patients during clinical studies, as well as to act as companion diagnostics for personalization of the treatment protocols by the physician^92^. As TMZ is an oral drug it allows for higher flexibility of time-of-administration especially when taken in an at-home setting^78^. Data from cellular models showed highest response rates for TMZ at the peak of *BMAL1*^48^. This opens avenues for clinical applications, since the peak of *BMAL1* could theoretically be shifted by external zeitgebers, such as artificial light conditions. This allows to adapt the circadian profile of a given patient to the appropriate clinical setting, thus facilitating treatment in real conditions fitted to the clinics’ daily routines^93^.

The same underlying idea may be applied to optimize and individualize the timing of radiotherapy. The only study conducted in humans so far compared morning vs. afternoon radiotherapy in 109 patients and found no significant differences in survival and adverse effects^81^, but this should not discourage from further research applying more detailed and complex study protocols.

Small-molecule CRY and REV-ERB agonist are likely to be a promising complement to the current standard of care therapy. Notably, in mice SR9009 showed comparable effects to TMZ, but with significantly reduced adverse effects^52^. This could mean that using such an agonist could reduce the amount of TMZ needed and therefore the adverse effects expected, especially relevant when optimizing the treatment time based on the individual clock profile of the patient as discussed in the previous section. Additionally, the clock proteins CRY2, PER1/2 and CLOCK were associated with regulating the response to irradiation. Radiosensitivity was shown to be highest at the peak of PER2 and the trough of CRY2 in rat models. Therefore, either using CRY/REV-ERB agonists to directly or indirectly target those clock proteins, or optimizing the time of day for irradiation, could be highly beneficial.

Interestingly, melatonin was shown to exert anti-tumour effects in GBM models and to decrease resistance to TMZ^1^. Therefore, melatonin may be another very exciting approach to aid applications of circadian medicine in GBM.

Tumour Treating Fields (TTF) are another exciting option to treat GBM. The EF-11 and EF-14 trials showed improved OS and PFS in newly diagnosed patients and improved quality of life in patients with recurrent GBM^94,95^. However, major disadvantages of this form of therapy are its high costs, its low availability in some countries and the stigma of wearing a visible scalp device. TTF is based on alternating electric fields and was shown to influence several processes including mitosis, migration, membrane permeability, DNA repair, autophagy and the immune system. TTF is very attractive as it shows high selectivity for cancer cells and low systemic toxicity^96^. Notably, the clock is also implicated in the processes regulated by TTF. As the mechanism of action of TTF is not yet fully understood, it remains to be elucidated if there are overlapping pathways between the clock and TTF in GBM, and if those therapeutic strategies can be combined. Notably, TTF was shown to improve the permeability of the BBB, and the cell membrane^96^, meaning that the pharmacokinetics of anti-tumour drugs will likely be altered when combining them with TTF. This has to be taken into account when timing drug administration.

The clock influences several important hallmarks and pathways in GBM. This implies that combining current emerging targeted therapies such as therapies targeting energy metabolism, angiogenesis, the PI3K/AKT/mTORC pathway^2^ or immune therapies^63,64^ with chronotherapy could improve their performance. Future research should therefore focus on exploring feasible methodologies to characterize the relationship between those GBM features and the clock in individual patients, and how to exploit it therapeutically.

### Concluding remarks

GBM is a highly aggressive and lethal form of brain cancer with limited treatment options. It remains a challenge in oncology due to its complex and heterogeneous nature, leading to rapid progression and poor outcomes for patients. Despite advances in treatment modalities, including surgery, radiation, and chemotherapy, the prognosis for GBM patients remains dismal. The molecular mechanisms underlying its pathogenesis involve the dysregulation of multiple signalling pathways, leading to the acquisition of hallmark cancer traits such as uncontrolled cell proliferation, evasion of apoptosis, and angiogenesis. However, as shown in this review, emerging evidence suggests that the circadian clock plays a role in GBM development and progression, potentially providing a new avenue for therapeutic interventions. Notably, targeting the molecular clock was shown to be selective to GBM, but not healthy brain cells^16,52^. Further research is needed to fully understand the intricate interplay between the circadian system and GBM pathophysiology. These promising results are likely to lead to the development of new therapeutic strategies targeting the circadian clock, and to the identification of new targets for the development of more effective treatments, providing hope for improved outcomes for patients with this devastating disease.

## Data Availability

No datasets were generated or analysed for this article.
